# Supplementary material for: NANOGP1, a tandem duplicate of NANOG, exhibits partial functional conservation in human naïve pluripotent stem cells
Source: Development. 2023 Jan 19;150(2):dev201155. doi: 10.1242/dev.201155 (PMC10110494; doi:10.1242/dev.201155)
Supplement: Supplementary information [file develop-150-201155-s1.pdf]

Figure S1

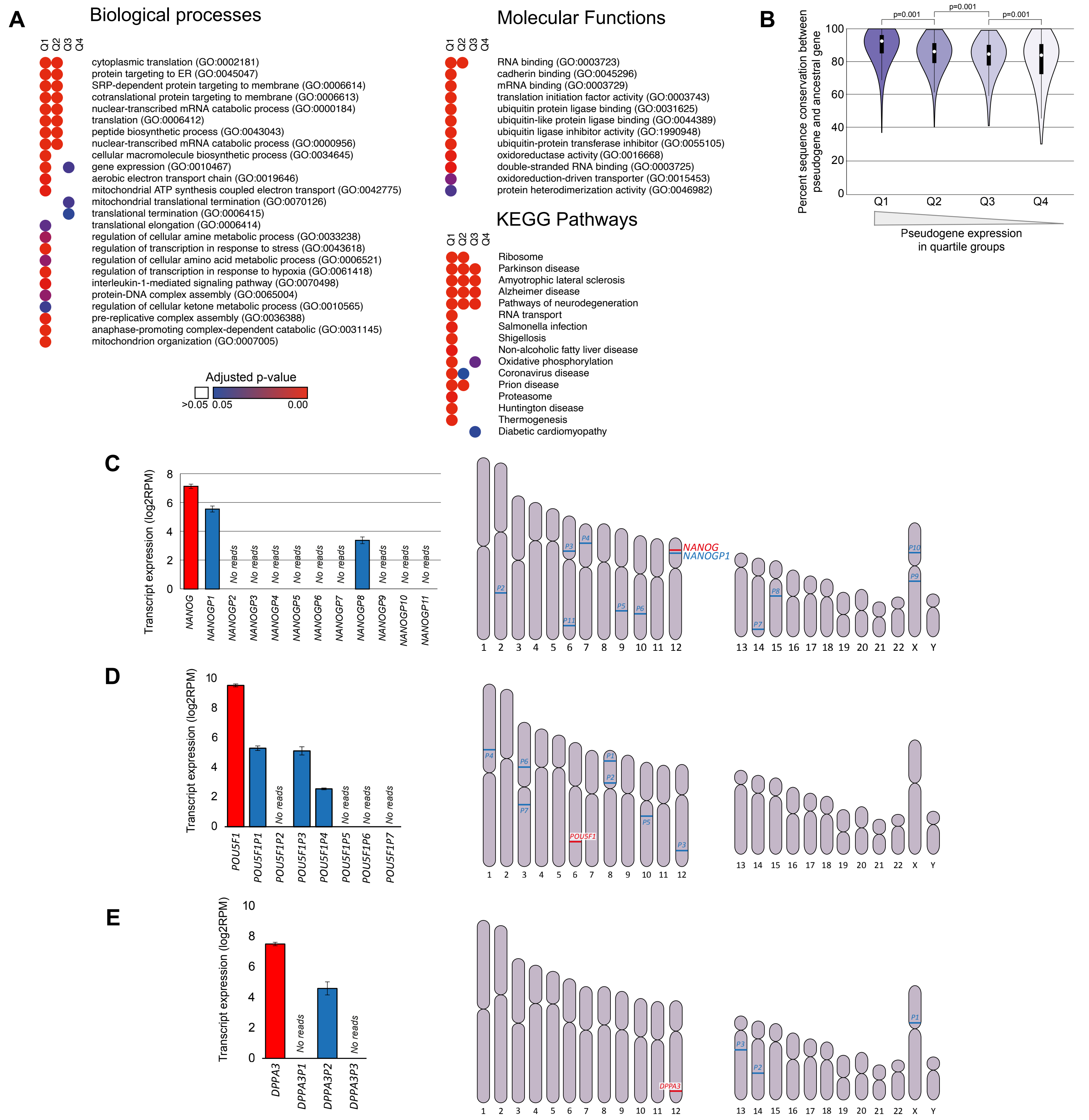

Fig. S1. Overview of pseudogenes and their ancestral genes

A) Gene ontology analysis of ancestral genes binned by the expression level in naive hPSCs of their associated pseudogenes. The highest expressed pseuogene was used for each ancestral gene. Q1, highest expressed quartile; Q4, lowest expressed quartile. There were 419 genes in each quartile group. Adjusted p-values were calculated using a Fisher's exact test. The analayis was performed using Enrichr (Chen et al., 2013; Kuleshov et al., 2016; Xie et al., 2021).
B) Violin plot of the percent sequence conservation between the coding sequence of the ancestral gene and the transcript sequence of the associated pseudogene. The pseudogenes were ranked by their expression level in naive hPSCs and divided into quartile groups (1300 pseudogenes per group). White circles, median; boxes, 25th and 75th percentiles; whiskers, 1.5 times the interquartile range. One way ANOVA (3 d.f.; 169.7 F), p=1.e-16. Post-hoc Tukey test reported p<0.005 for each pair-wise comparison.
C–E) *NANOG* (**C**), *POU5F1* (**D**) and *DPPA3* (**E**) transcript levels in naïve hPSCs (red) compared to the expression of their pseudogenes (blue). Values derived using a standard (Ensembl) annotation of genes and pseudogenes. Data show mean±s.d. from three biologically independent samples. Idiograms show the chromosomal locations of *NANOG* (**C**), *POU5F1* (**D**) and *DPPA3* (**E**) and their pseudogenes.

Development • Supplementary information

Figure S2

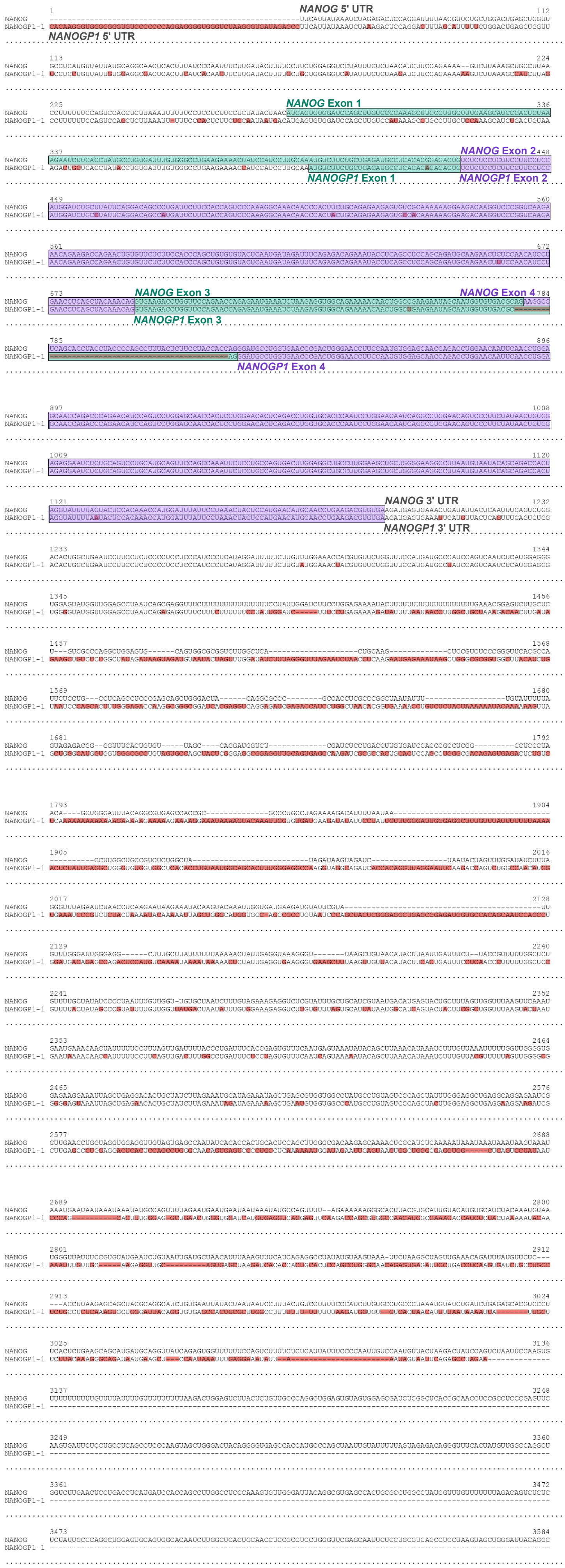

Fig. S2. Comparison of transcript sequence between *NANOG* and *NANOGP1*

NANOG and *NANOGP1* (isoform 1) mRNA sequence alignment. Exons are highlighted in green and purple. Sequence mismatches are highlighted in red. In total, the *NANOG* exon sequence contains 21 bp mismatches, and a 48bp deletion.

Development • Supplementary information

Figure S3

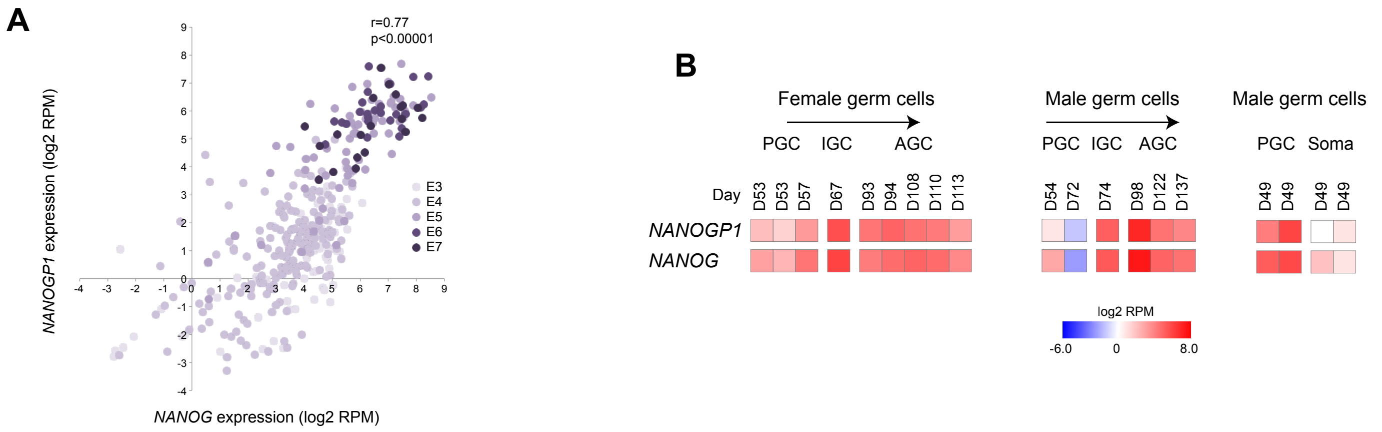

Fig. S3. *NANOGP1* expression in pluripotent cells in vivo

A) Scatter plot shows the expression of *NANOG* and *NANOGP1* expression in individual cells of the inner cell mass and epiblast lineages from embryonic day E3 to E7. Data were reanalysed from (Petropoulos et al., 2016).

B) Heat maps show *NANOG* and *NANOGP1* expression in human male and female germ cells over the indicated days of foetal development. PGC, primordial germ cells; IGC, intermediate germ cells; AGC, advanced germ cells. Bulk RNA-seq data were re-analysed from (Gkountela et al., 2015).

Figure S4

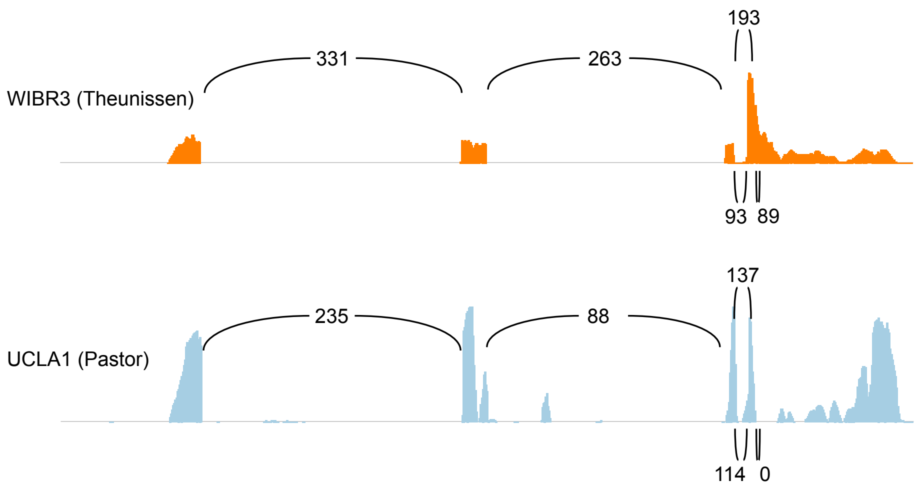

Fig. S4. Examina ion of *NANOGP1* in the genomes of non-human primates.

Sashimi plots show splicing analysis of*NANOGP1* transcripts in naïve hPSCs using RNA-seq data from two additional studies using different cell lines (Pastor et al., 2016; Theunissen et al., 2016). The numbers in between the RNA-seq peaks indicate the number of times a splicing event was measured. All of the individual data sets examined revealed that there are three different predicted patterns of transcript splicing.

Development • Supplementary information

Figure S5

A

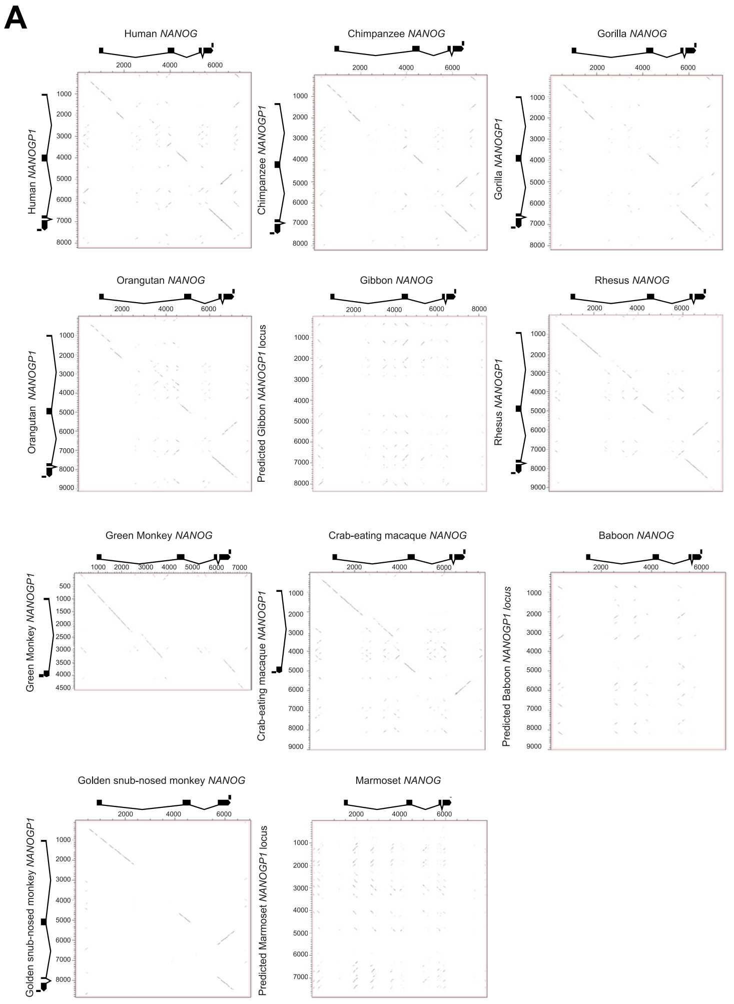

B

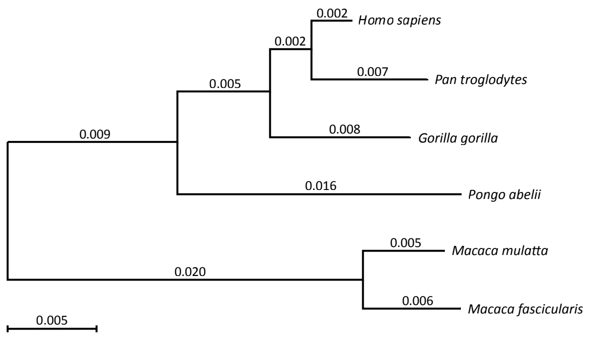

SEQUENCE DIVERGENCE MATRIX		*NANOGP1*					
		Homo sapiens	*Pan troglodytes*	*Gorilla gorilla*	*Pongo abelii*	*Macaca mulatta*	*Macaca fascicularis**
NANOG	*Homo sapiens*	0.0125	0.0190	0.0151	0.0254	0.0399	0.0487
	Pan troglodytes	0.0151	0.0164	0.0151	0.0257	0.0399	0.0414
	Gorilla gorilla	0.0151	0.0190	0.0075	0.0281	0.0412	0.0523
	Pongo abelii	0.0177	0.0190	0.0191	0.0164	0.0412	0.0380
	Macaca mulatta	0.0346	0.0386	0.0387	0.0412	0.0229	0.0345
	*Macaca fascicularis**	0.0346	0.0386	0.0387	0.0412	0.0229	0.0345

C

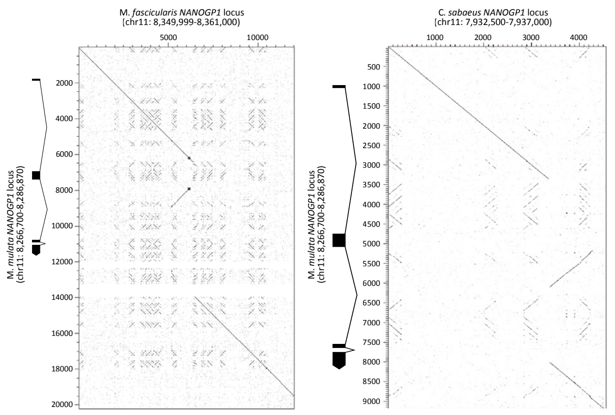

Fig. S5. Examina ion of *NANOGP1* in the genomes of non-human primates.
A) Dot plots show the alignment of primate *NANOG* orthologs to their corresponding *NANOGP1* duplicates. Individual dots represent matching base pairs between the two aligned sequences. In areas of sequence conservation, individual dots form diagonal lines. Gene/pseudogene structure is shown as rectangles (exons) and lines (introns). Scale, bp.
B) Upper, phylogenetic tree based on *NANOGP1* coding sequence. Neighbour-joining tree was based on the maximum likelihood model. Numbers on branches indicate evolutionary distance and correspond to substitutions/sequence length ratios. Substitutions are defined as nucleotides that are different from human *NANOGP1*. Lower, pairwise sequence divergence rates (# of substitutions/sequence length) of *NANOG* and *NANOGP1* coding sequences. Numbers correspond to substitutions per sequence length ratio. *In M. fascicularis genome, only 1st and 2nd exons are present.
C) Dotter plots show partial *NANOGP1* deletions in green monkey and crab-eating macaque genomes.

Development • Supplementary information

Figure S6

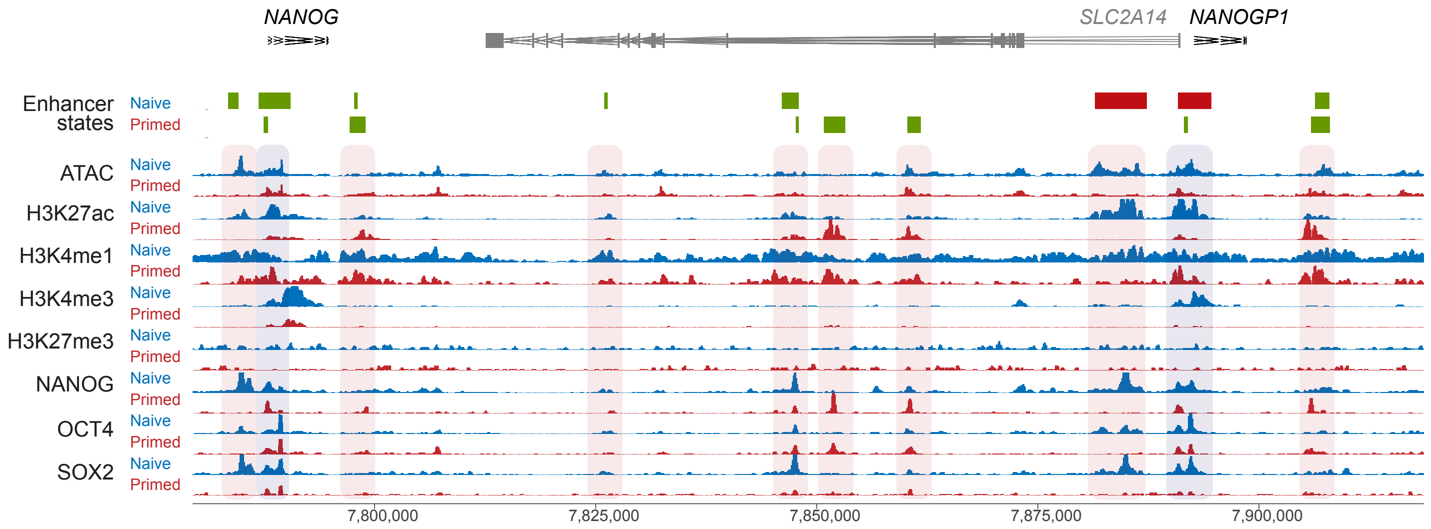

Fig. S6. Characterisation of *NANOGP1* putative regulatory sequences.

Genome browser tracks of ATAC-seq (Pastor et al., 2016) and ChIP-seq (Chovanec et al., 2021) profiles across the *NANOG/ NANOGP1* locus in naïve and primed hPSCs. The enhancer state tracks indicate the positions of previously defined enhancers (green boxes) and super-enhancers (red boxes) in each cell type; annotations from (Chovanec et al., 2021).

Development • Supplementary information

Figure S7

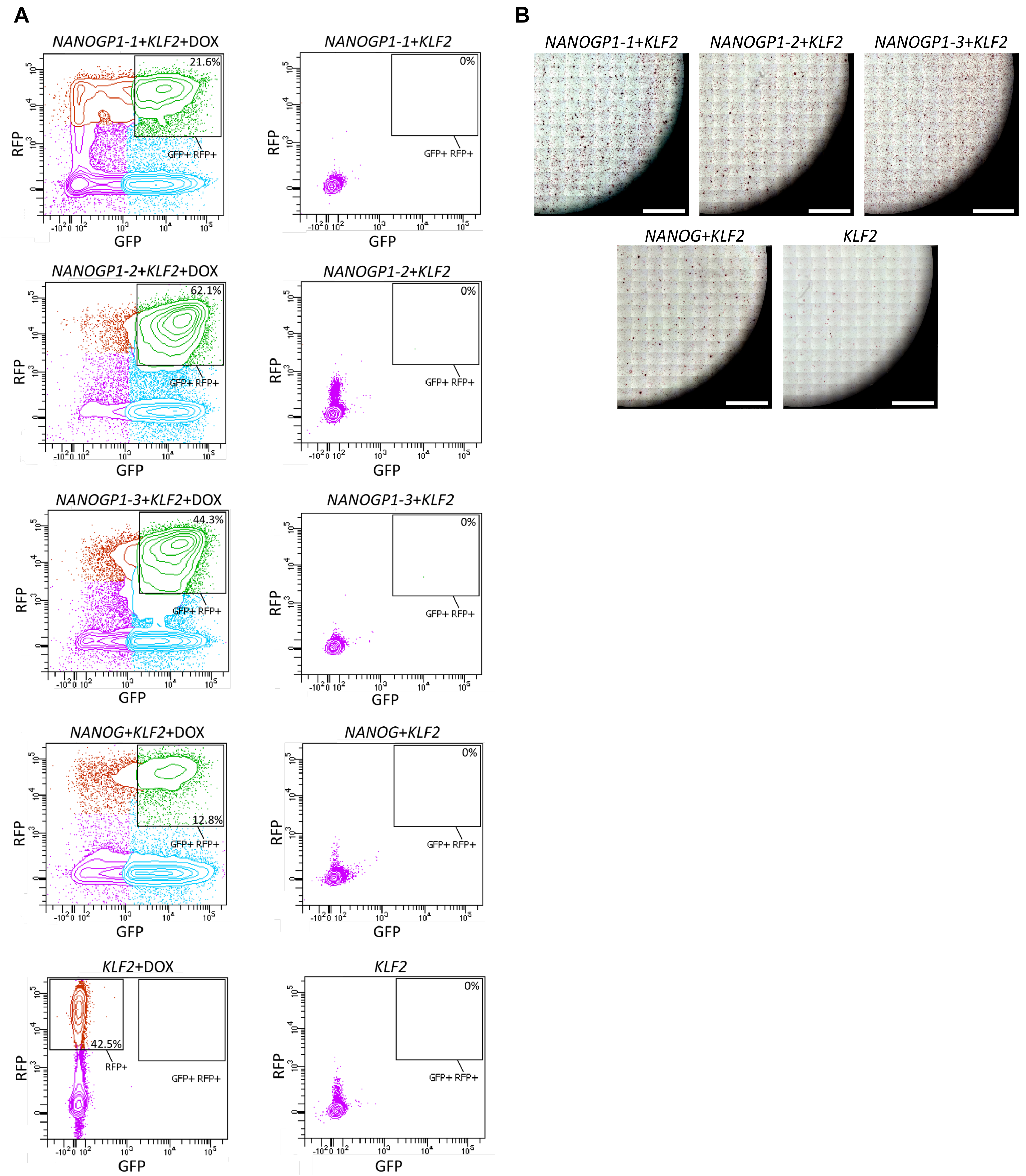

Fig. S7. Characterisation of transgene-induced primed to naïve hPSC reprogramming.
A) Flow cytometry contour plots show RFP and GFP expression in transgenic primed hPSCs. Samples treated with DOX for 48 h are shown on the left; non-treated samples on the right. Percentages of GFP+RFP+ and RFP+ populations are indicated. Data are representative of three biologically independent experiments.
B) Brightfield microscopy images of the alkaline phosphatase assay. Reprogrammed naïve hPSC colonies are stained in purple. Scale, 5 mm.

Development • Supplementary information

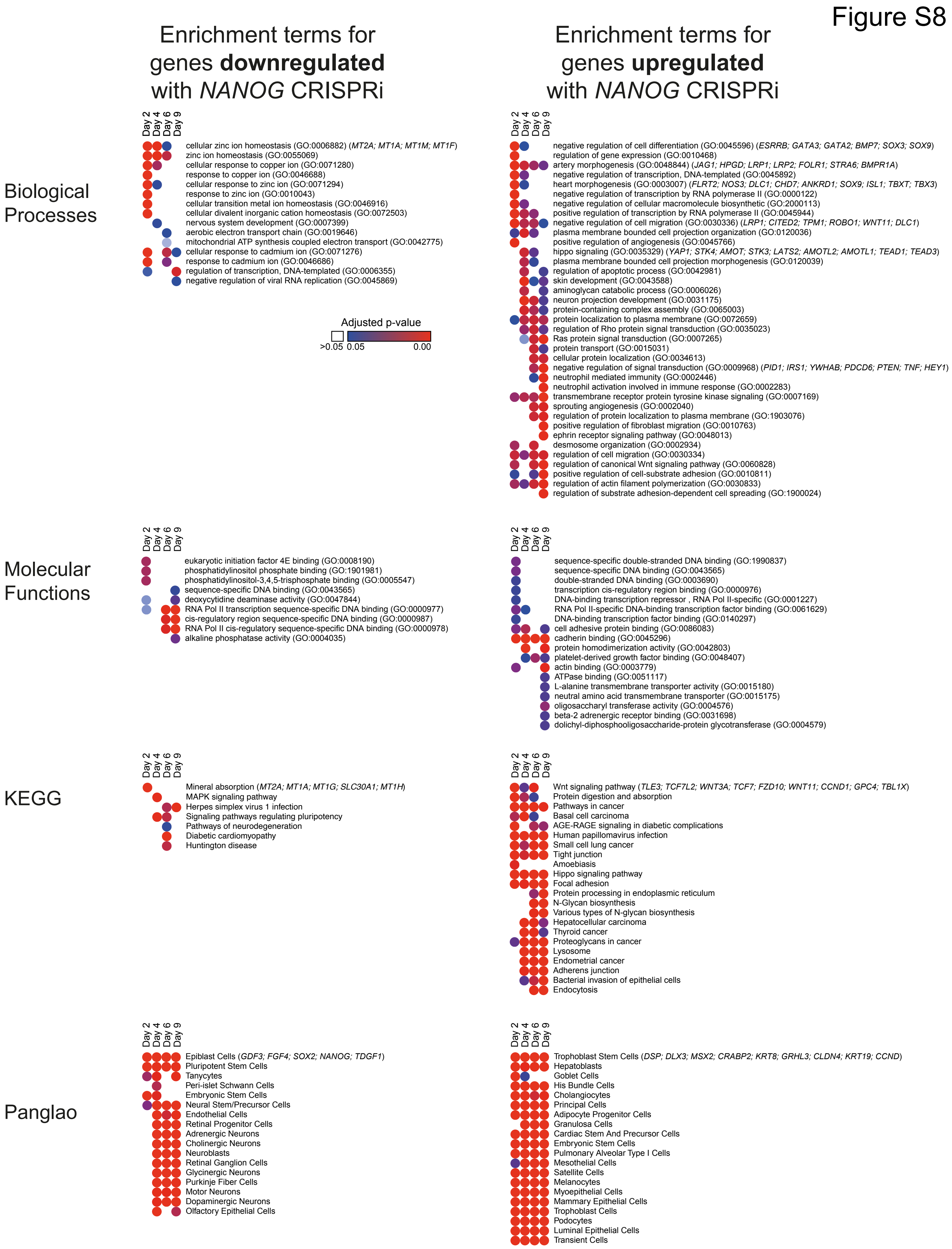

Fig. S8. Gene ontology analysis of differentially expressed genes following *NANOG* CRISPRi in naive hPSCs. Gene ontology analysis of genes significantly downregulated (left) or upregulated (right) following induction of *NANOG* CRISPRi in naive hPSCs at days 2, 4, 6 and 9. Adjusted p-value calculated using Fisher's exact test. The analayis was performed using Enrichr (Chen et al., 2013; Kuleshov et al., 2016; Xie et al., 2021).

Development • Supplementary information

Table S1. Optimisation of *in vivo* DNA cutting efficiency for *NANOGP1* epitope tag insertion.

**Experiment**	**gRNA / crRNA sequence**	***In vivo* DNA cutting efficiency (% PCR amplicons with deletions per sample)**
NANOGP1 5′ Cas9 #1	GGCCCACAAATCACAGGTAT	18%
NANOGP1 5′ Cas9 #2	GAGATGCCTCACACAGAGAC	15%
NANOGP1 5′ Cas12a	TGGGCCTGAAGAAAACCATCC	70%
NANOGP1 3′ Cas9	GATGTTACTCAGTTTCAGTC	9%
NANOG 5′ Cas9	CCTCTATACTAACATGAGTG	30%
NANOG 3′ Cas9	GATATTACTCAATTTCAGTC	3%

Table S2. ssODN templates used in the *NANOGP1* epitope tagging experiment.

AS – antisense strand; S – sense strand. Tag sequence is in bold. Homology arms are in capital letters.

**ssODN name**	**ssODN sequence, 5′-3′**
NANOGP1_3xFLAG_AS	TTACCAGTCTCTGTGTGAGGCATCTCAGCAGAAGACATTTGCAAGGATGG**cttgtcatcgtcatccttgtaatcgatgtcatgatctttataatcaccgtcatggtctttgtagtc**CATATGGTTTTCTTCAGGCCCACAAATCACAGGTATAGGTGACCAGTCTTTAC
NANOGP1_V5_S	GTAAAGACTGGTCACCTATACCTGTGATTTGTGGGCCTGAAGAAAACCATATG**ggtaagcctatccctaaccctctcctcggtctcgattctacg**CCATCCTTGCAAATGTCTTCTGCTGAGATGCCTCACACAGAGACTGGTAA

Table S3. attB primer sequences used for generating TetON hPSC lines.

attB sequences are in bold.

**Primer name**	**Primer sequence**
5′-attB-NANOGP1-F	**GGGGACAAGTTTGTACAAAAAAGCAGGCTCT**ATGTCTTCTGCTGAGATGCC
5′-attB-NANOG-F	**GGGGACAAGTTTGTACAAAAAAGCAGGCTCT**ATGAGTGTGGATCCAGCTTG
5′-attB-NANOGP1/NANOG-R	**GGGGACCACTTTGTACAAGAAAGCTGGGTC**TCACACGTCTTCAGGTTGC
5′-attB-KLF2-F	**GGGGACAAGTTTGTACAAAAAAGCAGGCTCT**ATGGCGCTGAGTGAACCC
5′-attB-KLF2-R	**GGGGACCACTTTGTACAAGAAAGCTGGGTC**CTACATGTGCCGTTTCATGTGC

Development • Supplementary information

Table S4. Primers for the pgRNA-CKB gRNA cloning.

TTGG and AAAC in bold are the overhangs added to clone phospho-annealed oligonucleotides to pgRNA-CKB using *BsmBI* restriction.

Primer name	Primer sequence (5'-3')
NANOGP1-gRNA-F	**TTGG**TGAGTCGCCTCCACAATAAC
NANOGP1-gRNA-R	**AAAC**GTTATTGTGGAGGCGACTCA
NANOG-gRNA-F	**TTGG**CCAGCAGAACGTTAAAATCC
NANOG-gRNA-R	**AAAC**GGATTTTAACGTTCTGCTGG

Table S5. Western Blotting and protein immunoprecipitation antibodies. WB, Western Blotting; N/A, not applicable.

Target	Conjugation	Reactivity	Host	Dilution	Clone	Company	Cat. #	Batch	Docs
IgG	HRP	Mouse	Goat	1:10000	Poly.	BioRad	1706516	–	–
IgG	HRP	Rabbit	Goat	1:10000	Poly.	BioRad	1706515	–	–
IgG	HRP	Goat	Rabbit	1:10000	Poly.	BioRad	1721034	–	–
IgG	Dylight 680	Mouse	Donkey	1:10000	Poly.	Cell Signalling	5470	TF2580476	Link
IgG	Dylight 800	Rabbit	Donkey	1:10000	Poly.	Cell Signalling	5151	TI2633311	Link
FLAG	None	N/A	Mouse	1:10000	M-2	Sigma Aldrich	F3165	SLBQ7119V	Link
NANOG	None	Human	Rabbit	1:1000	Poly.	Abcam	AB21624	GN3206521-7	Link
NANOG	None	Human	Goat	1:1000	Poly.	R&D	AF1997	KKJ0922031	Link
V5	None	N/A	Rabbit	1:1000	DBH8Q	Cell Signalling	13202	6	Link

Table S6. Immunofluorescent staining antibody details.

CST, Cell Signalling Technology; SC, Santa Cruz; TFS - ThermoFisher Scientific.

Target	Conjugate	Reactivity	Host	Dilution	Clone	Company	Cat. #	Batch	Docs
IgG	AF555	Goat	Donkey	1:1000	Polyclonal	TFS	A21432	2026158	Link
IgG	AF647	Mouse	Donkey	1:1000	Polyclonal	TFS	A31571	2136787	Link
IgG	AF555	Rabbit	Donkey	1:1000	Polyclonal	TFS	A31572	2339822	Link
NANOG	None	Human	Goat	1:200	Polyclonal	R&D	AF1997	KKJ0922031	Link
OCT4	None	Human/ mouse	Mouse	1:300	C-10	SC	SC5279	D1119	Link
V5	None	N/A	Rabbit	1:150	DBH8Q	CST	13202	6	Link

Development • Supplementary information

Table S7. Flow cytometry antibodies.

Dilution ratios per 100 µl buffer per 500,000 cells.

Target	Conjugation	Reactivity	Dilution	Clone	Company	Cat. #	Batch	Docs
CD24	BUV395	Human	1:80	ML5 RUO	BD Biosciences	563818	8295751	Link
CD75	eF660	Human	1:40	LN-1	eBioscience	50-0759-42	22861107	Link
CD77	PE-CF594	Human	1:40	5B5	BD Biosciences	563631	8288944	Link
Cd90.2	APC-Cy7	Mouse	1:40	30-H12	BioLegend	105328	B307867	Link
Viability Dye	eF780	N/A	1:33	N/A	eBioscience	65-0865-18	N/A	Link
SSEA4	APC	Human/ mouse	1:50	MC-813-70	R&D	FAB1435A	LTB6919101	Link
SUSD2	PE	Human	1:200	REA795	Miltenyi Biotec	130-111-641	REA795	Link
SUSD2	FITC	Human	1:20	W5C5	Miltenyi Biotec	130-127-935	5200800890	Link
SUSD2	BV421	Human	1:200	W5C5	BD Biosciences	749533	316894	Link

Table S8. Primers used for genotyping, cloning validation and Sanger sequencing.

F, R; forward and reverse primer orientation.

Primer name	Assay	Primer sequence (5'-3')
M13-20-F	Sanger Sequencing; genotyping	GTAAAACGACGGCCAGT
M13-R	Sanger Sequencing; genotyping	CATGGTCATAGCTGTTTCC
attL1-F	Sanger Sequencing; genotyping	CTACAAACTCTTCCTGTTAGTTAG
attL2-R	Sanger Sequencing; genotyping	ATGGCTCATAACACCCCTTG
pgRNA-CKB-F	Sanger Sequencing	GAGATCCAGTTTGGTTAGTACCGGG
pgRNA-CKB-R	Sanger Sequencing	ATGCATGGCGGTAATACGGTTAT
*NANOGP1*_7/5'-F	Sanger sequencing; genotyping	TCCTGTTATTGTGGAGGCGA
FLAG-R	Genotyping	TGGCTTGTCATCGTCATCCT
V5-R	Genotyping	GGAGAGGGTTAGGGATAGGC
P1-tag-seq-F	Sanger Sequencing	GATCCAGCTTGTCCATAAAGCC

Development • Supplementary information

Table S9. RT-qPCR primer sequences.

Gene	Forward primer sequence (5'-3')	Reverse primer sequence (5'-3')
DPPA3	AGACCAACAAACAAGGAGCCT	CCCATCCATTAGACACGCAGA
GFP	CTTCAAGATCCGCCACAACATC	GGGTGCTCAGGTAGTGGTTGTC
HMBS	AGGAGTTCAGTGCCATCATCCT	CACAGCATACATGCATTCCTCA
NANOG endogenous	CCACTTTCTTGCACAGACCA	CTGGAGTTGCTGGCAGAAAG
NANOG_1	CTTGTCCCCAAAGCTTGCCT	AGGCCCACAAATCACAGGCA
NANOG_2	AAGCATCCGACTGTAAAGAATCT	ACATTTGCAAGGATGGATAGT
NANOGP1_1	CTTGTCCATAAAGCCTGCCT	AGGCCCACAAATCACAGGTA
NANOGP1_2	AAGCATCTGACTGTAAAGACTGG	ACATTTGCAAGGATGGATGGT
OCT4	GGATATACACAGGCCGATGTGG	ATGGTCGTTTGGCTGAATACCT
TFCP2L1	TTTGTGGGACCCTGCGAAG	TGCTTAAACGTGTCAATCTGGA

Table S10. Primate genome assemblies used in the evolutionary analyses.

Species	Assembly	First release date
Human	GRCh38	2013
Chimpanzee	panTro6	2018
Bonobo	panPan2	2015
Gorilla	gorGor5	2016
Orangutan	ponAbe3	2018
Gibbon	*nomLeu3*	*2012*
Crab-eating macaque	macFas5	2013
Rhesus macaque	rheMac8	2015
Marmoset	calJac3	2009

Development • Supplementary information
