# Supplementary material for: NANOGP1, a tandem duplicate of NANOG, exhibits partial functional conservation in human naïve pluripotent stem cells
Source: Development. 2023 Jan 19;150(2):dev201155. doi: 10.1242/dev.201155 (PMC10110494; doi:10.1242/dev.201155)
Supplement: Supplementary file 1 [file develop-150-201155_review_history.pdf]

NANOGP1, a tandem duplicate of *NANOG*, exhibits partial functional conservation in human naive pluripotent stem cells

Katsiaryna Maskalenka, Gökberk Alagöz, Felix Krueger, Joshua Wright, Maria Rostovskaya, Asif Nakhuda, Adam Bendall, Christel Krueger, Simon Walker, Aylwyn Scally and Peter J. Rugg-Gunn
DOI: 10.1242/dev.201155

Editor: Maria Elena Torres-Padilla

Review timeline

Original submission:	26 July 2022
Editorial decision:	25 August 2022
First revision received:	14 December 2022
Accepted:	16 December 2022

Original submission

First decision letter

MS ID#: DEVELOP/2022/201155

MS TITLE: NANOGP1, a tandem duplicate of NANOG, exhibits partial functional conservation in human naive pluripotent stem cells

AUTHORS: Kate Maskalenka, Gokberk Alagoz, Felix Krueger, Joshua Wright, Maria Rostovskaya, Asif Nakhuda, Adam Bendall, Christel Krueger, Simon Walker, Aylwyn Scally, and Peter Rugg-Gunn

I have now received all the referees reports on the above manuscript, and have reached a decision. The referees' comments are appended below, or you can access them online: please go to BenchPress and click on the 'Manuscripts with Decisions' queue in the Author Area.

As you will see, the overall evaluation is very positive and we would like to publish a revised manuscript in Development, provided that the referees' comments can be satisfactorily addressed. Please attend to all of the reviewers' comments in your revised manuscript and detail them in your point-by-point response. If you do not agree with any of their criticisms or suggestions explain clearly why this is so. If it would be helpful, you are welcome to contact us to discuss your revision in greater detail. Please send us a point-by-point response indicating your plans for addressing the referee's comments, and we will look over this and provide further guidance.

Reviewer 1

Advance summary and potential significance to field

The manuscript "NANOGP1, a tandem duplicate of NANOG, exhibits partial functional conservation in human naïve pluripotent stem cells" by Maskalenda et al., deals with an interesting aspect of evolution, gene duplications and the functional relevance of pseudogenes. After a short characterization of pseudogene expression in pluripotent stem cells, the authors characterize one particular example, NANOGP1, which has been classified as an unprocessed pseudogene of the pluripotency factor NANOG. The paper is well written the experiments are plausible and the figures informative.

© 2023. Published by The Company of Biologists under the terms of the Creative Commons Attribution License (https://creativecommons.org/licenses/by/4.0/).

The topic of pseudogene relevance in general, but also particularly of those pseudogenes relating to pluripotency factors, has been to some degree controversial, making studies investigating this issue highly interesting for a broader scientific community. It is still under debate, how frequently those pseudogenes are expressed in pluripotent cells, how well they can be distinguished from another, to which degree those are translated to stable proteins, whether those proteins are in principle capable of conducting cellular functions and finally, whether they also have a role under physiological conditions. The manuscript at hand contains a series of interesting experiments concerning NANOGP1 that provide evidence for each of these questions. However, some clarifications are necessary to judge how strong these indications are, particularly concerning evidence and strength of NANOGP1 protein expression under physiological conditions.

Comments for the author

Main points:
1. The characterization of expressed pseudogenes in pluripotent cells is highly interesting, it would be informative to characterize the functional roles of the origin genes (GO terms etc) and the mutational load, relative to expression status and class (processed/unprocessed).

2. One key achievement of the manuscript at hand is a strategy to clearly assign sequencing reads to NANOG or NANOGP1. “Although NANOGP1 is a duplicated copy of NANOG, there were sufficient sequence differences between the transcripts of the two genes to uniquely assign RNA-seq reads to each gene (Sequence Divergence Rate of 0.013). We also confirmed that NANOG reads do not map to the NANOGP1 locus and vice versa when using 12 a high mapping quality value (MAPQ>20).” It would be highly informative for the reader to learn a little bit more about that, best in form of a figure:

Where in the genes are the sequence differences? How many are there? How many reads are assigned clearly (based on how many differences)? How many reads cannot be clearly assigned? Could sequencing-/amplification errors be ruled out in most cases?

3. The Tagging of NANOGP1 with protein Tags is a straight forward strategy to proof protein expression during physiological condition. Several aspects of this and the following experiments are however unclear: (a) Can the tagged protein be detected in immunoblots (without enrichment through IP or overexpression)? If not, what is the estimate of protein levels compared to NANOG? (b) The main justification classifying NANOGP1 as a pseudogene is based on the canonical ATG not producing a functional protein, the main argument of the authors is based on the assumption that another one, a second downstream ATG is used. However, the protein tags are on the amino-terminal side and bring their own sequence environment and ATG into the mix. Did the authors produce a C-terminal Tag, was this detectable?

4. The results derived from overexpression of NANOGP1 are overall convincing. In this condition the protein seems to be easily detected via immunoblot, indicating much higher protein levels. Can the authors compare/estimate overexpressed vs endogenous protein levels of NANGP1?

5. Is there evidence from published IP-MS (against NANOG or co-factors), that not only NANOG, but also NANOGP1 has been detected, or are there technical reasons that it could not have been found/distinguished?

Small points:

1. The finding that pseudogenes seem to be particularly frequent for pluripotency genes seems interesting, but it is unclear, whether this is statistically correct and whether it might be explained by most of those genes being expressed in the germ line, where retrotransposition has to take place (at least for processed pseudogenes).

2. Maybe the authors could make sure datasets analysed are connected to an original citation in the main text (and not just in methods or figure).

© 2023. Published by The Company of Biologists under the terms of the Creative Commons Attribution License (https://creativecommons.org/licenses/by/4.0/).

3. The proposed NANOGP1 protein misses 39 AA. Could the authors speculate a bit more, based on published genetic experiments and resolved/computed structures, what the function of these AA might be?

4. Immunoflourescence against the V5 Tag is a meaningful experiment. It is however unclear, what is shown in Figure 4C. Is this a colony containing four cells? How do bigger colonies look, is there a salt and pepper distribution of expression or strictly positive and negative colonies?

5. Since all three NANOGP1 isoforms work in the reprogramming assay it would be informative to get more information about, how the differences relate to proposed NANOG functions.

Reviewer 2

Advance summary and potential significance to field

The manuscript by Maskalenka et al focuses on pseudogenes of key human pluripotency factors. It centers on a duplication of NANOG in human development, revealing that the previously known pseudogene NANOGP is in fact protein coding gene that promotes ground state human pluripotency. Also, it shows that the duplication of NANOG took place in primate evolution much earlier than previously thought, and that it became dysfunctional in old world monkeys, while retaining integration in the pluripotency circuitry of pluripotency in apes. Overall, the manuscript provides ample data about human pluripotency pseudogenes, and creating many tools to further investigate NANOGP which are very useful. It is an impressive paper, meticulously performed, and it fits very well to Development journal. I recommend accepting it as is, and would only recommend to slightly rephrase the introduction (also the abstract) which currently gives a slightly misleading impression that the focus of the paper is only human NONOGP (especially last paragraph of introduction), while in fact the paper has a much broader breadth, which elegantly investigates and discusses the evolution of NANOG genes. I would also recommend describing the isoforms of NANOGP in Apes if possible from existing databases, as it could be interesting to review the divergence of NANOGP isoforms reflecting on function. But this is not a must, and the paper presents a very nice clear story that is a pleasure to read. I strongly recommend acceptance to Development.

Comments for the author

recommend to rephrase the introduction (also the abstract) which currently gives a slightly misleading impression that the focus of the paper is only human NONOGP (especially last paragraph of introduction), while in fact the paper has a much broader breadth, which elegantly investigates and discusses the evolution of NANOG genes. I would also recommend describing the isoforms of NANOGP in Apes if possible from existing databases, as it could be interesting to review the divergence of NANOGP isoforms reflecting on function.

Reviewer 3

Advance summary and potential significance to field

Maskalenka et al. investigate the evolution and functional conservation of NANOGP1, a tandem duplicate of NANOG, in naïve human pluripotent stem cells (hPSC). While gene duplication events are important drivers of evolution, the role of pseudogenes in naïve pluripotency and early mammalian development remains poorly characterized. Through careful bioinformatic analysis, the authors observe that several key pluripotency factors have highly expressed pseudogenes in naïve hPSCs. They focus their attention on NANOGP1, since it is unprocessed, located within the same locus as its ancestral copy, and also highly expressed in the human pre-implantation epiblast. Evolutionary genetic analysis reveals that NANOGP1 gene and protein sequences are highly conserved in the Great Apes, but inactivated in other non-primate species via different alterations. NANOGP1 also retains putative regulatory sequences, including a promoter region that shows enrichment of SOX2 and H3K4me3 peaks in the naive state. The authors go on to demonstrate that NANOGP1 is in fact a protein-coding gene and retains some of the unique functional properties of its ancestral copy such as the capacity to autorepress endogenous NANOG and reprogram primed

© 2023. Published by The Company of Biologists under the terms of the Creative Commons Attribution License (https://creativecommons.org/licenses/by/4.0/).

hPSCs to naïve pluripotency upon co-expression with KLF2. On the other hand disruption of NANOGP1 using an inducible CRISPRi approach resulted in fewer gene expression changes compared to disruption of NANOG, suggesting that NANOGP1 is dispensable for the maintenance of naïve pluripotency.

This is an interesting and technically rigorous study that reveals how gene duplication can contribute to transcription factor activity in pluripotent cells. As the authors point out, the fact that NANOGP1 promotes reprogramming but is dispensable for its maintenance is reminiscent of another transcription factor, KLF17, as recently shown by Kathy Niakan's lab. This manuscript is suitable for publication in Development with minor revisions, although I would encourage the authors to consider several suggestions for future work.

Comments for the author

1. In Fig. 4D, why was NANOGP1 not recognized in the input lane using the NANOG C-terminal antibody, which targets the conserved region of the protein. Is the expression of this duplicated copy too low to be detected by standard western blot?

2. The authors show that overexpression of NANOGP1 results in downregulation of endogenous NANOG in naïve hPSCs, indicating that NANOGP1 shares the capacity for autorepression with NANOG (Fig. 5). I wonder whether this autorepression is unique to the context of naïve pluripotency or also seen in primed hPSCs?

3. Inducible CRISPRi reveals a clear contrast between knocking down NANOG vs. NANOGP1: NANOG CRISPRi resulted in upregulation of various trophoblast markers but NANOGP1 CRISPRi did not (Fig. 7E). Can the authors explain why GATA2 and GATA3 are upregulated during this time course in the untreated NANOG CRISPRi cells?

4. The scatter plots and PCA in Fig. 7F-G indicate that NANOGP1 CRISPRi results in far fewer gene expression changes compared to NANOG CRISPRi. It would be instructive to include a more detailed analysis on the DEGs after NANOG depletion, for example by performing gene ontology and gene set enrichment analyses. Based on the embryo alignments in Fig. 7H, NANOG CRISPRi induces a shift towards TE/CTB fate, but there may be effects on other pathways that are not reflected in this analysis.

5. Since NANOGP1 is specifically enriched in the naïve state, I wonder whether the authors could test whether its overexpression affects the ability of naïve cells to undergo the naïve-to-primed transition? This could be investigated by comparing the ability of NANOG O/E and NANOGP1 O/E naïve cells to be re-adapted to primed media or undergo capacitation into a formative state (Rostovskaya et al., 2019). A plausible hypothesis is that forced expression of NANOGP1 will delay the upregulation of primed markers.

Minor comments:

- Fig. S1A-C: the legend indicates that pseudogenes are shown in yellow, but they're actually shown in blue

- p. 21, line 16: please change "has" to "have" in the sentence: the dynamics of the transcriptional response following NANOG perturbation, and the effect on gene expression programmes, [have] not been examined

- p. 25, line 19: please add "it" in the sentence: to such an extent that [it] was only recently recognised as a duplicate of NANOG

- p. 26, line 3: please change "are" to "is" in the sentence: Careful annotation of pseudogenes, ideally supported by functional data, [is] important

© 2023. Published by The Company of Biologists under the terms of the Creative Commons Attribution License (https://creativecommons.org/licenses/by/4.0/).

First revision

Author response to reviewers' comments

We are delighted and very grateful to have received such constructive and positive comments from all three reviewers. We respond below to each point raised. Changes made to the revised manuscript are highlighted in blue text in the accompanying file.

Please note that in addition to the changes described below, we have also updated Fig. 1B to correct a labelling error that we spotted in the previous figure. The message of the new figure remains the same as before.

Reviewer 1 Advance Summary and Potential Significance to Field:
The manuscript "NANOGP1, a tandem duplicate of NANOG, exhibits partial functional conservation in human naïve pluripotent stem cells" by Maskalenda et al., deals with an interesting aspect of evolution, gene duplications and the functional relevance of pseudogenes. After a short characterization of pseudogene expression in pluripotent stem cells, the authors characterize one particular example, NANOGP1, which has been classified as an unprocessed pseudogene of the pluripotency factor NANOG. The paper is well written, the experiments are plausible and the figures informative.

The topic of pseudogene relevance in general, but also particularly of those pseudogenes relating to pluripotency factors, has been to some degree controversial, making studies investigating this issue highly interesting for a broader scientific community. It is still under debate, how frequently those pseudogenes are expressed in pluripotent cells, how well they can be distinguished from another, to which degree those are translated to stable proteins, whether those proteins are in principle capable of conducting cellular functions and finally, whether they also have a role under physiological conditions. The manuscript at hand contains a series of interesting experiments concerning NANOGP1 that provide evidence for each of these questions. However, some clarifications are necessary to judge how strong these indications are, particularly concerning evidence and strength of NANOGP1 protein expression under physiological conditions.

Reviewer 1 Comments for the Author:

Main points:

1. The characterization of expressed pseudogenes in pluripotent cells is highly interesting, it would be informative to characterize the functional roles of the origin genes (GO terms etc) and the mutational load, relative to expression status and class (processed/unprocessed).

We have completed this analysis and present the new results in Figs. S1A and S1B. We ranked the expression of pseudogenes in naive pluripotent stem cells and binned the associated ancestral genes into quartile groups (419 genes per group). For each ancestral gene, we used the highest expressed pseudogene. Because the vast majority of the pseudogenes are processed (~95%) we retained both processed and unprocessed pseudogenes. We then examined GO and KEGG terms for the genes in each expression quartile cohort. We found that the ancestral genes of the most highly expressed pseudogenes (quartile 1) were specifically enriched for functions related to RNA binding and protein translation (Fig. S1A). Genes in the lowest expression quartiles were not significantly enriched for any GO or KEGG terms.

We also examined the mutational load and found an interesting connection between the expression levels of pseudogenes and the sequence conservation with their ancestral gene (Fig. S1B). To do this, we ranked pseudogenes based on their expression levels in naive hPSCs and binned the pseudogenes into quartile groups. We then calculated the sequence conservation between the coding sequence of the ancestral gene and the transcript sequence of the pseudogene (we chose this approach because most pseudogenes are processed, and also because few coding sequences have been annotated for pseudogenes). Overall, the more highly expressed the pseudogene, the greater the sequence conservation. We have added this result to the main text and to Fig. S1B.

© 2023. Published by The Company of Biologists under the terms of the Creative Commons Attribution License (https://creativecommons.org/licenses/by/4.0/).

2. One key achievement of the manuscript at hand is a strategy to clearly assign sequencing reads to NANOG or NANOGP1. “Although NANOGP1 is a duplicated copy of NANOG, there were sufficient sequence differences between the transcripts of the two genes to uniquely assign RNA- seq reads to each gene (Sequence Divergence Rate of 0.013). We also confirmed that NANOG reads do not map to the NANOGP1 locus and vice versa when using a high mapping quality value (MAPQ>20).” It would be highly informative for the reader to learn a little bit more about that, best in form of a figure: Where in the genes are the sequence differences? How many are there? How many reads are assigned clearly (based on how many differences)? How many reads cannot be clearly assigned? Could sequencing/amplification errors be ruled out in most cases?

In the revised manuscript, we include a new figure that shows the number and position of the sequence differences between *NANOG* and *NANOGP1* transcripts (Fig. S2).

When examining the RNA-seq read assignments, we performed the following: We used the cDNA sequences of *NANOG* (*NANOG-201* transcript) and *NANOGP1* (isoform 1 transcript) to simulate FastQ files as 100bp single-end reads in 1bp steps from start to end. These *NANOG* and *NANOGP1* files were aligned to both the default GRCh38 genome and a custom GRCh38 *NANOGP1* genome with HISAT2 with default parameters; this was done to determine whether adding the new *NANOGP1* annotation made a difference. To remove reads that align to several positions in the genome (multi-mapping reads) we applied a mapping quality (MAPQ) filter of 20 or greater (unique alignments); no filtering (MAPQ = 0) allows both unique as well as multi-mapping alignments to be kept for visualisation purposes. This gives a readout of how many reads are assigned and not assigned in each condition.

When importing sequence reads into SeqMonk software with a MAPQ filter of > 20 to remove ambiguously aligning sequences, the NANOG-201 cDNA aligns nicely to exons in *NANOG*, and there is no cross-mapping of *NANOGP1* reads at all (Reviewer Figure 1, left). Adding the *NANOGP1* annotation does not make an obvious difference (Reviewer Figure 1, top). Furthermore, *NANOGP1* reads map nicely to the *NANOGP1* locus, with the *NANOGP1* annotation adding a small number of extra reads (Reviewer Figure 1, right). There is no noteworthy cross- mapping of *NANOG* reads to the *NANOGP1* locus (Reviewer Figure 1, right). This also suggests that sequencing and amplification errors are negligible.

If we allow reads to multimap (MAPQ = 0), this added some extra reads and also resulted in a few reads cross-mapping from *NANOG* to *NANOGP1*, and vice versa, mostly in regions that appear unmappable in the MAPQ20 setting. Using 43bp long reads, as in the Petropoulos et al. data set, results in a similar picture, even though the reduced read length resulted in a lower mapping rate overall.

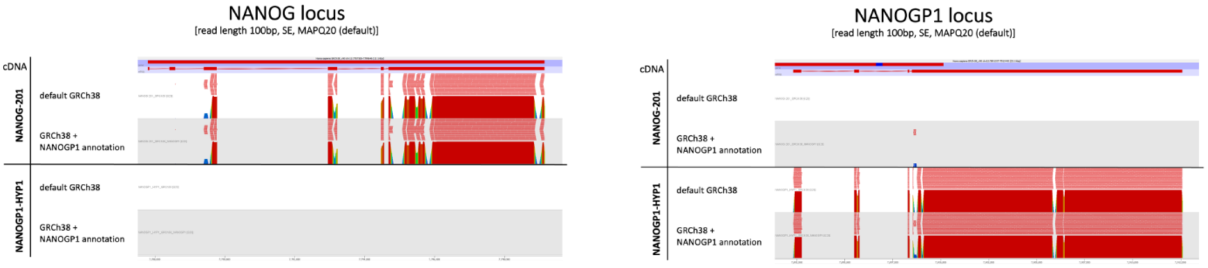

Reviewer Figure 1: Visualisation of simulated RNA-seq reads to the *NANOG* and *NANOGP1* loci. Left: Genome browser screenshot of the *NANOG* locus, showing the mapping of simulated *NANOG* (NANOG-201) or *NANOGP1* (NANOGP1-HYP1) RNA-seq reads. No *NANOGP1* reads align to the *NANOG* locus with the standard mapping parameters used (MAPQ20).
Right: A similar analysis shows that very few simulated *NANOG* reads map to the *NANOGP1* locus.

3. The Tagging of NANOGP1 with protein Tags is a straight forward strategy to proof protein expression during physiological condition. Several aspects of this and the following experiments are however unclear: (a) Can the tagged protein be detected in immunoblots (without enrichment through IP or overexpression)? If not, what is the estimate of protein levels compared to NANOG? (b) The main justification classifying NANOGP1 as a pseudogene is based on the canonical ATG not

© 2023. Published by The Company of Biologists under the terms of the Creative Commons Attribution License (https://creativecommons.org/licenses/by/4.0/).

producing a functional protein, the main argument of the authors is based on the assumption that another one, a second downstream ATG is used. However, the protein tags are on the amino-terminal side and bring their own sequence environment and ATG into the mix. Did the authors produce a C-terminal Tag, was this detectable?

(a) We cannot detect the tagged protein in immunoblots without enrichment. We believe this is because the proportion of cells with the epitope tag is low in the population. Because of this, we cannot compare or estimate the protein levels between NANOG and NANOGP1. However, we believe that NANOG is certainly the more abundant protein.

(b) We have tried to target the C-terminal region of NANOGP1, but unfortunately were unsuccessful. The sequences are identical between *NANOG* and *NANOGP1* near to the stop codon, so this makes it very difficult to design gene-specific gRNAs. The nearest potential gRNA site is 34bp from the stop codon, which is a distance at which homology-directed repair is very unlikely to occur. Nevertheless, we tested and compared the cutting efficiency of several gRNAs/crRNAs near to both the start and stop codons of *NANOGP1*. We provide a new Table S1 to show the cutting efficiencies and we now mention this in this main text. The results show that the cutting efficiencies near to the stop codon of *NANOGP1* (and *NANOG*) are very low, with only ~9% of amplicons in the target area being cut. This poor efficiency is too low for successful homology-directed repair and epitope tag integration. In contrast, sequences targeting the start codon of *NANOGP1* had a cutting efficiency of ~70%, which was high enough to enable successful homology-directed repair.

4. The results derived from overexpression of NANOGP1 are overall convincing. In this condition the protein seems to be easily detected via immunoblot, indicating much higher protein levels. Can the authors compare/estimate overexpressed vs endogenous protein levels of NANGP1?

Because we cannot detect endogenous protein without enrichment, we are unable to compare or estimate the levels of overexpressed vs endogenous NANOGP1. We have looked at this for NANOG using Western blot densitometry analysis, and we estimate that there is a four-fold increase in NANOG protein levels when overexpressed. It is possible that it might be similar for NANOGP1 but we cannot be confident about this.

5. Is there evidence from published IP-MS (against NANOG or co-factors), that not only NANOG, but also NANOGP1 has been detected, or are there technical reasons that it could not have been found/distinguished?

Thank you for this suggestion. A curious (and frustrating) feature of NANOG is that it is poorly detected by typical mass spectrometry analysis. For example, NANOG was not detected in two recent proteomic experiments in human naive pluripotent stem cells that examined chromatin-bound proteins (Zijlmans et al 2022, PMID: 35697783) and whole cell proteins (Di Stefano et al 2018, PMID: 30127506), yet many other transcription factors were abundantly detected.

We have now looked at this more closely to understand why. It seems as though NANOG has a particularly unfavourable distribution of trypsin cleavage sites, which means that trypsin digest would yield a few very large peptides and a small number of very small peptides, which would not be detected in a typical mass spectrometry analysis. At best, there would be seven detectable NANOG peptides. Of these, NANOGP1 shares four of them. Moreover, there are no additional cleavage sites in NANOGP1 that are missing in NANOG, meaning that unambiguous detection of NANOGP1 is very challenging. We were able to detect peptides that correspond to NANOG (QPTSAEK) and NANOGP1 (QPTTAEK), but because they are so short, they give very few fragments, which in a highly complex background is not robust enough to be conclusive at this point. Interestingly, one peptide reportedly corresponding to NANOGP1 was detected in the Di Stefano et al dataset. The abundance of this peptide was 5-10 fold higher in naive cells compared to primed, and this difference was consistent in three different human pluripotent stem cell lines. This difference would be consistent with our data, although without knowing the sequence of the NANOGP1 peptide in the published dataset we cannot be sure that it corresponds unambiguously to NANOGP1 and not also to NANOG.

© 2023. Published by The Company of Biologists under the terms of the Creative Commons Attribution License (https://creativecommons.org/licenses/by/4.0/).

Small points:

1. The finding that pseudogenes seem to be particularly frequent for pluripotency genes seems interesting, but it is unclear, whether this is statistically correct and whether it might be explained by most of those genes being expressed in the germ line, where retrotransposition has to take place (at least for processed pseudogenes).

Thank you - it is good to clarify this. Although GO terms related to “stem cells” are significantly enriched in the list of ancestral genes (adjusted p-value 0.002), it is not one of the top ranked terms and so we would prefer to take a more cautious position and not emphasise this point. We realise that our phrase “In particular..” in the original manuscript was misleading, so we have removed that text from the revised manuscript.

We agree with the reviewer that the ancestral genes of processed pseudogenes must be expressed in cells that contribute to the germ line including epiblast cells and germ cells, and therefore it is expected that this category of gene/pseudogene might be enriched for terms related to stem cells and germ cells. Whether genes that are highly expressed in epiblast and the germ line are also predisposed to duplication events is an interesting and unresolved question. We include this in the discussion and have added “and the germ line” to make this point clearer.

2. Maybe the authors could make sure datasets analysed are connected to an original citation in the main text (and not just in methods or figure).

Done

3. The proposed NANOGP1 protein misses 39 AA. Could the authors speculate a bit more, based on published genetic experiments and resolved/computed structures, what the function of these AA might be?

We have added new text to the discussion on this point. The 39 amino acid deletion does not overlap with any domains that are known to be responsible for *NANOG*-specific functions. Predicted protein structures suggest the N-terminus of NANOG is largely unstructured. However, several studies have investigated the potential function of the N-terminal domain. For instance, Chang and colleagues proposed a *NANOG* bivalency model (Chang et al., 2009, PMID: 19350681), in which the NANOG N-terminus was hypothesised to oppose the transactivation role of the C-terminus. Indeed, deleting the entire 122 amino acid N-terminal domain led to a significant increase in transactivation activity mediated by the remaining HD-CR1-WR-CR2 domains. This was demonstrated by two separate research groups, in Chang et al., 2009 and Oh et al., 2005, by testing ΔND-HD-CR1-WR-CR2 luciferase assay constructs in non-human primates and human cell lines. This, however, has not been tested directly in hPSCs. To investigate this topic further, the 39 amino acid deletion would have to be performed in the same way as the 122 amino acid deletion was performed in these previous studies.

Another potential function for the NANOG N-terminus was suggested by Oh and colleagues, who hypothesised that it could be required for post-translational protein modifications, such as phosphorylation and ubiquitination (Oh et al., 2005, PMID: 16000880). The human NANOG N-terminal domain contains 11 phosphorylation sites (Brumbaugh et al., 2014, PMID: 24678451; Ho et al., 2012, PMID: 22493428; Wang et al., 2019, PMID: 30595535; Xie et al., 2014, PMID: 23708658), and one of them, Y35, is within the 39 amino acid deletion and so is absent from the predicted NANOGP1 protein isoforms. The consequences of deleting Y35 in NANOG in hPSCs are currently unknown. However, in cancer cells, substituting Y35 with T inhibited its interaction with a tyrosine kinase FAK, involved in regulation of cell migration, and ultimately prevented the formation of the expected filopodia-formation phenotype in NANOG overexpression studies (Ho et al., 2012). This could imply that in hPSCs, the absence of just one this phosphorylation site could be sufficient to impair one or more downstream phosphorylation pathways.

In summary, based on our proposed protein structure for NANOGP1, the NANOGP1 N-terminal deletion could hypothetically affect its downstream phosphorylation pathways involved in ubiquitination and protein turn over. Similarly, the N-terminal deletion could cause altered co-

© 2023. Published by The Company of Biologists under the terms of the Creative Commons Attribution License (https://creativecommons.org/licenses/by/4.0/).

repressor binding. However, both of these models would require additional protein-protein interaction assays in hPSCs to be examined.

4. Immunoflourescence against the V5 Tag is a meaningful experiment. It is however unclear, what is shown in Figure 4C. Is this a colony containing four cells? How do bigger colonies look, is there a salt and pepper distribution of expression or strictly positive and negative colonies?

Yes, the image in Fig. 4C shows a colony of four naive cells that are all positive for the tag. We have modified the figure legend to make this clearer. Elsewhere in the field of view, there are small colonies that are negative for the epitope tag. As we could not select for the correct integration of the tag, we opted to examine the cells when the colonies were fairly small in case the untagged cells outcompeted the tagged cells. We have therefore not examined later stage cultures that might have contained larger colonies. The small colonies typically contained cells that were all either positive or all negative, and rarely showed a mixed expression pattern.

5. Since all three NANOGP1 isoforms work in the reprogramming assay it would be informative to get more information about, how the differences relate to proposed NANOG functions.

All three predicted NANOGP1 isoforms contain intact homeodomains that are identical in sequence to the NANOG homeodomain, and we have adjusted the manuscript text to make this clearer. As NANOG's homeodomain is sufficient to reprogramme mouse cells to a pluripotent state (Theunissen et al., PMID: 22028025) then it makes sense that NANOGP1 is also a potent inducer of naive pluripotency. The tryptophan repeats and C-terminal domains are also conserved; these regions are thought to provide the capacity of NANOG with the ability to homodimerise and also heterodimerise with other proteins, such as SOX2 (e.g. Gagliardi et al., PMID: 23892456; Mullin et al., PMID: 27939294). An interesting future direction is to test the prediction that NANOGP1 protein should also be capable of forming homo- and heterodimers and to ask questions about whether this might disrupt or augment NANOG-containing dimers.

As the predicted isoforms of NANOGP1 are highly similar to each other, there are few differences in which to further understand the functional regions of NANOG. Instead, all NANOGP1 isoforms diverge from NANOG in the N-terminal, where NANOGP1 lacks the first 39 amino acids. As NANOGP1 can fulfil several of the main functions of NANOG, including reprogramming and gene autorepression then we are able to conclude that the missing N-terminal sequence is not required for these roles. In the manuscript, we have expanded the discussion of what the potential consequences could be for the N-terminal truncation.

Reviewer 2 Advance Summary and Potential Significance to Field:
The manuscript by Maskalenka et al focuses on pseudogenes of key human pluripotency factors. It centers on a duplication of NANOG in human development, revealing that the previously known pseudogene NANOGP is in fact protein coding gene that promotes ground state human pluripotency. Also, it shows that the duplication of NANOG took place in primate evolution much earlier than previously thought, and that it became dysfunctional in old world monkeys, while retaining integration in the pluripotency circuitry of pluripotency in apes. Overall, the manuscript provides ample data about human pluripotency pseudogenes, and creating many tools to further investigate NANOGP which are very useful. It is an impressive paper, meticulously performed, and it fits very well to Development journal. I recommend accepting it as is, and would only recommend to slightly rephrase the introduction (also the abstract) which currently gives a slightly misleading impression that the focus of the paper is only human NANOGP (especially last paragraph of introduction), while in fact the paper has a much broader breadth, which elegantly investigates and discusses the evolution of NANOG genes. I would also recommend describing the isoforms of NANOGP in Apes if possible from existing databases, as it could be interesting to review the divergence of NANOGP isoforms reflecting on function. But this is not a must, and the paper presents a very nice clear story that is a pleasure to read. I strongly recommend acceptance to Development.

Reviewer 2 Comments for the Author:
Recommend to rephrase the introduction (also the abstract) which currently gives a slightly misleading impression that the focus of the paper is only human NANOGP (especially last paragraph of introduction), while in fact the paper has a much broader breadth, which elegantly

© 2023. Published by The Company of Biologists under the terms of the Creative Commons Attribution License (https://creativecommons.org/licenses/by/4.0/).

investigates and discusses the evolution of NANOG genes. I would also recommend describing the isoforms of NANOGP in Apes if possible from existing databases, as it could be interesting to review the divergence of NANOGP isoforms reflecting on function.

Thank you for the good suggestion - we have rephrased the abstract and introduction to include the new findings on *NANOGP1* evolution.

We have tried to investigate *NANOGP1* isoforms in apes but unfortunately this has proved to be very difficult. The main limitation is that there are no published RNA-seq data sets in apes from cell types in which *NANOGP1* is highly expressed (such as early embryos cells, or naive-state pluripotent stem cells). The closest available data sets in apes are from primed-state pluripotent stem cells but, as anticipated from our earlier analysis of human *NANOGP1*, these samples lack sufficient reads across the *NANOGP1* locus that are needed to confidently identify exon-exon splicing and other basic features of *NANOGP1* isoforms in apes.

Reviewer 3 Advance Summary and Potential Significance to Field:
Maskalenka et al. investigate the evolution and functional conservation of NANOGP1, a tandem duplicate of NANOG, in naïve human pluripotent stem cells (hPSC). While gene duplication events are important drivers of evolution, the role of pseudogenes in naïve pluripotency and early mammalian development remains poorly characterized. Through careful bioinformatic analysis, the authors observe that several key pluripotency factors have highly expressed pseudogenes in naïve hPSCs. They focus their attention on NANOGP1, since it is unprocessed, located within the same locus as its ancestral copy, and also highly expressed in the human pre-implantation epiblast. Evolutionary genetic analysis reveals that NANOGP1 gene and protein sequences are highly conserved in the Great Apes, but inactivated in other non-primate species via different alterations. NANOGP1 also retains putative regulatory sequences, including a promoter region that shows enrichment of SOX2 and H3K4me3 peaks in the naive state. The authors go on to demonstrate that NANOGP1 is in fact a protein-coding gene and retains some of the unique functional properties of its ancestral copy, such as the capacity to autorepress endogenous NANOG and reprogram primed hPSCs to naïve pluripotency upon co-expression with KLF2. On the other hand, disruption of NANOGP1 using an inducible CRISPRi approach resulted in fewer gene expression changes compared to disruption of NANOG, suggesting that NANOGP1 is dispensable for the maintenance of naïve pluripotency.

This is an interesting and technically rigorous study that reveals how gene duplication can contribute to transcription factor activity in pluripotent cells. As the authors point out, the fact that NANOGP1 promotes reprogramming but is dispensable for its maintenance is reminiscent of another transcription factor, KLF17, as recently shown by Kathy Niakan's lab. This manuscript is suitable for publication in Development with minor revisions, although I would encourage the authors to consider several suggestions for future work.

Reviewer 3 Comments for the Author:

1. In Fig. 4D, why was NANOGP1 not recognized in the input lane using the NANOG C-terminal antibody, which targets the conserved region of the protein. Is the expression of this duplicated copy too low to be detected by standard western blot?
Yes, we believe that NANOGP1 protein levels are low, especially compared to NANOG. Enriching endogenous NANOGP1 by epitope-tag pulldown concentrates the protein to levels that are detectable by western blot using an anti-NANOG antibody (C-terminal).

2. The authors show that overexpression of NANOGP1 results in downregulation of endogenous NANOG in naïve hPSCs, indicating that NANOGP1 shares the capacity for autorepression with NANOG (Fig. 5). I wonder whether this autorepression is unique to the context of naïve pluripotency or also seen in primed hPSCs?

We have performed this experiment and found that gene autorepression is also seen in primed hPSCs, so this feature is not unique to the context of naive-state pluripotency (new Fig. 5E). We performed this experiment by overexpressing *NANOGP1* in primed hPSCs. The results show that the endogenous expression levels of both *NANOG* and *NANOGP1* are reduced following ectopic

© 2023. Published by The Company of Biologists under the terms of the Creative Commons Attribution License (https://creativecommons.org/licenses/by/4.0/).

NANOGP1 induction. Although the cell states are not exactly equivalent, this finding is in line with the reported result that mouse *Nanog* exhibits gene autorepression in mouse PSCs cultured in both 2i+LIF and serum+LIF conditions (Navarro et al., PMID: 23178592).

3. Inducible CRISPRi reveals a clear contrast between knocking down NANOG vs. NANOGP1: NANOG CRISPRi resulted in upregulation of various trophoblast markers, but NANOGP1 CRISPRi did not (Fig. 7E). Can the authors explain why GATA2 and GATA3 are upregulated during this time course in the untreated NANOG CRISPRi cells?

We are also puzzled by this and do not have a particularly good explanation. We think that the induction of some trophoblast markers, such as *GATA2* and *GATA3*, is because the cells by days 6 to 9 of the experiment are starting to become a bit overgrown or are undergoing some other stress-related effect that might be destabilising the cells towards the end of the timecourse. It could also be that the CRISPRi plasmid has some leakiness in these conditions that could exacerbate the upregulation of these two genes. Nevertheless, the transcriptomes of untreated *NANOG* CRISPRi cells at all timepoints closely overlap with one another and also with epiblast cells (Fig. 5H) and so we believe the upregulation of *GATA2* and *GATA3* is not indicative of more substantial induction of trophoblast programmes.

4. The scatter plots and PCA in Fig. 7F-G indicate that NANOGP1 CRISPRi results in far fewer gene expression changes compared to NANOG CRISPRi. It would be instructive to include a more detailed analysis on the DEGs after NANOG depletion, for example by performing gene ontology and gene set enrichment analyses. Based on the embryo alignments in Fig. 7H, NANOG CRISPRi induces a shift towards TE/CTB fate, but there may be effects on other pathways that are not reflected in this analysis.

We have completed the suggested analysis and the new results are shown in Fig. S8 and discussed in the main text. We examined gene ontology and gene enrichment terms in genes that are either downregulated or upregulated at each timepoint following the induction of *NANOG* CRISPRi. Genes downregulated following *NANOG* CRISPRi are associated predominantly with pluripotent stem cells, epiblast cells and with specific gene ontology terms, such as metal ion homeostasis (*MT2A*, *MTS1A*). Upregulated genes are enriched for trophoblast stem cell factors, mesoderm development (*CHD7*, *ISL1*, *SOX9*, *TBXT*, *GATA6*) and Hippo and Wnt signalling pathways. Trophoblast regulators dominate the transcriptional profiles and are therefore given the primary focus in the manuscript. However, the induction of some mesoderm-associated genes is of potential interest as it raises new thoughts about the exit of naive pluripotency and the specification of additional lineages during early post-implantation development, potentially including mesenchymal cells and amnion (that express some genes that are shared with mesoderm cell types).

5. Since NANOGP1 is specifically enriched in the naïve state, I wonder whether the authors could test whether its overexpression affects the ability of naïve cells to undergo the naïve-to-primed transition? This could be investigated by comparing the ability of NANOG O/E and NANOGP1 O/E naïve cells to be re-adapted to primed media or undergo capacitation into a formative state (Rostovskaya et al., 2019). A plausible hypothesis is that forced expression of NANOGP1 will delay the upregulation of primed markers.

We have completed this experiment and the new results are shown in Fig. 6G-I. The data support the reviewer's hypothesis that forced expression of *NANOGP1* delays the upregulation of primed markers during capacitation, as assessed by flow cytometry (CD24 and SSEA4) and by RT-qPCR (*DUSP6*). One caveat to note is that the endogenous level of *NANOG* is rapidly downregulated following *NANOGP1* induction (consistent with the repressive activity that we have demonstrated earlier in the manuscript) and so we believe that the defective naive to primed capacitation might also be partly explained by elevated cell differentiation due to *NANOG* downregulation. Furthermore, we observed a significant increase in cell death following *NANOGP1* overexpression particularly at the later stages of capacitation.

© 2023. Published by The Company of Biologists under the terms of the Creative Commons Attribution License (https://creativecommons.org/licenses/by/4.0/).

Minor comments:

- Fig. S1A-C: the legend indicates that pseudogenes are shown in yellow, but they're actually shown in blue.

- p. 21, line 16: please change "has" to "have" in the sentence: the dynamics of the transcriptional response following NANOG perturbation, and the effect on gene expression programmes, [have] not been examined.

- p. 25, line 19: please add "it" in the sentence: to such an extent that [it] was only recently recognised as a duplicate of NANOG.

- p. 26, line 3: please change "are" to "is" in the sentence: Careful annotation of pseudogenes, ideally supported by functional data, [is] important

Thank you - all corrected.

Second decision letter

MS ID#: DEVELOP/2022/201155

MS TITLE: NANOGP1, a tandem duplicate of NANOG, exhibits partial functional conservation in human naive pluripotent stem cells

AUTHORS: Kate Maskalenka, Gokberk Alagoz, Felix Krueger, Joshua Wright, Maria Rostovskaya, Asif Nakhuda, Adam Bendall, Christel Krueger, Simon Walker, Aylwyn Scally, and Peter Rugg-Gunn
ARTICLE TYPE: Research Article

Thank you for submitting the revised version of the above manuscript, in which all of the Reviewers comments have been satisfactorily addressed. I am happy to tell you that your manuscript has been accepted for publication in Development, pending our standard ethics checks.

© 2023. Published by The Company of Biologists under the terms of the Creative Commons Attribution License (https://creativecommons.org/licenses/by/4.0/).
